# 3D Fabrication and Characterisation of Electrically Receptive PCL-Graphene Scaffolds for Bioengineered In Vitro Tissue Models

**DOI:** 10.3390/ma15249030

**Published:** 2022-12-17

**Authors:** Mary Josephine McIvor, Fionn Ó Maolmhuaidh, Aidan Meenagh, Shahzad Hussain, Gourav Bhattacharya, Sam Fishlock, Joanna Ward, Aoife McFerran, Jonathan G. Acheson, Paul A. Cahill, Robert Forster, David J. McEneaney, Adrian R. Boyd, Brian J. Meenan

**Affiliations:** 1 Nanotechnology and Integrated Bioengineering Centre (NIBEC), School of Engineering, Ulster University, 2-24 York Street, Belfast BT15 1AP, UK; 2 The National Centre for Sensor Research, School of Chemical Sciences, Dublin City University, Glasnevin, Dublin 9, Ireland; 3 School of Biotechnology, Dublin City University, Glasnevin, Dublin 9, Ireland; 4 Cardiovascular Research Unit, Craigavon Area Hospital, 68 Lurgan Road, Portadown, Co., Armagh BT63 5QQ, UK

**Keywords:** polycaprolactone, graphene, 3D fabrication, fused deposition modelling, bioactivity, electroactivity

## Abstract

Polycaprolactone (PCL) is a well-established biomaterial, offering extensive mechanical attributes along with low cost, biocompatibility, and biodegradability; however, it lacks hydrophilicity, bioactivity, and electrical conductivity. Advances in 3D fabrication technologies allow for these sought-after attributes to be incorporated into the scaffolds during fabrication. In this study, solvent-free Fused Deposition Modelling was employed to fabricate 3D scaffolds from PCL with increasing amounts of graphene (G), in the concentrations of 0.75, 1.5, 3, and 6% (*w*/*w*). The PCL+G scaffolds created were characterised physico-chemically, electrically, and biologically. Raman spectroscopy demonstrated that the scaffold outer surface contained both PCL and G, with the G component relatively uniformly distributed. Water contact angle measurement demonstrated that as the amount of G in the scaffold increases (0.75–6% *w*/*w*), hydrophobicity decreases; mean contact angle for pure PCL was recorded as 107.22 ± 9.39°, and that with 6% G (PCL+6G) as 77.56 ± 6.75°. Electrochemical Impedance Spectroscopy demonstrated a marked increase in electroactivity potential with increasing G concentration. Cell viability results indicated that even the smallest addition of G (0.75%) resulted in a significant improvement in electroactivity potential and bioactivity compared with that for pure PCL, with 1.5 and 3% exhibiting the highest statistically significant increases in cell proliferation.

## 1. Introduction

Three-dimensional (3D) fabrication is a fast-emerging technology for a range of biomedical engineering applications. Progression towards 3D-fabricated biomaterials (scaffolds) with added attributes, such as electrical conductivity, bioactivity, and biomimicry, offers the means to produce bioengineered electrically responsive tissue with increasing native-like structure and function [1]. Research continues into enhancing these attributes to better represent key aspects of real tissues for repair and regeneration as well as for the creation of new tissue models to study physiological events and diseases and to test new interventions [2,3].

In 1986, the first patent was submitted on 3D fabrication, also known as 3D printing, additive manufacturing, or rapid prototyping. In 2015, the ISO/ASTM 52900 standard was created to regularise terminology and classify each of the different types of 3D printers [4]. At present, 3D fabrication falls into seven categories: (i) Material Extrusion, to include fused deposition modelling (FDM), (ii) In situ Polymerisation, (iii) Powder Bed Fusion, (iv) Material Jetting, (v) Binder Jetting, (vi) Direct Energy Deposition, and (vii) Sheet Lamination [4]. FDM is the most commonly employed type for the additive manufacturing of thermoplastic components given its effectiveness and simplicity [5].

To create functional tissue constructs, precursor cells must be able to adhere, proliferate, and mature as if in their native in vivo environment. Part of this representative environment is a physical substrate (a scaffold) that is manufactured by a specific process using a specific material or combination of materials to a specific design, for example, with appropriately sized micro pores to maximise cell adhesion, proliferation, and maturation. Collectively, these mechanical, physico-chemical, electrical, and biological attributes allow a scaffold to best mimic the native-like environment of the target cell/tissue. Advances in 3D fabrication technologies offer a way to incorporate these sought-after attributes into scaffolds fabricated from appropriate biomaterials. The thermoplastic polycaprolactone (PCL) is a widely used biomaterial in bioengineering applications, both in research and industry, including its use as a 3D printing material, to create mechanically robust, thermally excluded 3D structures due to its low cost, bioinertness, biocompatibility, and biodegradability. Previous studies by Wang et al., Caetano et al., and Porta et al. reported the use of 3D-fabricated PCL-based scaffolds for bone tissue engineering applications [6,7,8]. In terms of engineering electrically excitable tissue, such as cardiac tissue, the literature suggests that scaffold fabrication is dominated by other fabrication technologies, namely Electrospinning Technology, such as that by Hitscherich et al. who applied electrospun graphene (G)-loaded PCL scaffolds for cardiac tissue engineering applications [9]. Whereas electrospinning has attributes for producing tissue constructs for repair and regeneration, 3D extrusion offers a means to create the variety of specialist component parts needed for advanced in vitro tissue models, such as cell-laden hydrogels and their support structures. The value of PCL in such models, especially those that will support electrically responsive cells, is limited by its poor hydrophilicity, bioactivity, and most importantly, electrical activity. In the case of an insulating polymer such as PCL, there is a need to provide this function via the inclusion of a conductive additive [10]. Graphene (G) and G-derivatives are a common addition to polymers to enhance their electroactivity in biomedical applications; Wang et al. added pure G pellets to pure PCL at 0.25, 0.50, and 0.75% (*w*/*w*) and fabricated scaffolds with a defined 3D lattice geometry, finding that the addition of G had a positive impact on cell response [6], and Bahrami et al. prepared electroconductive polyurethane/G nanocomposites using multi-layer G nanoflakes at 0.1, 2, 5, and 10% (*w*/*w*) and found a significant increase in electroactivity upon addition of G, along with surface topography and cell response [11].

In this study, material extrusion via FDM was employed to create 3D scaffolds due to its affordable, durable, and small footprint of the system. Three-dimensional scaffolds were prepared using solvent-free PCL and PCL+G inks by mixing appropriate amounts of each component in their as-received powder states with a heated extruder barrel to achieve melt-blending prior to extrusion. This approach allowed for the inclusion of higher G concentrations, i.e., in the range 0.75–6% (*w*/*w*), compared with that used in other published works; Hitscherich et al. and Ginestra employed a solvent (acetone and cyclopentatone, respectively) along with heat, sonication, and proficient stirring, i.e., solution-blending, prior to manufacture to thoroughly disperse G within PCL and, in doing so, were able to use G only at lower concentrations (0.005–0.05% and 1–2%) [9,12].

In this study, predetermined closed structure PCL scaffolds with increasing amounts of electroactive G up to 6% (*w*/*w*) were fabricated using FDM 3D printing. Scaffold potential to support electrically responsive cells was determined using physico-chemical characterisation, along with electrical characterisation by electrochemical impedance spectroscopy (EIS) of the pure PCL and PCL+G scaffolds to confirm the inclusion and influence of additives designed into them. Additionally, biological characterisation was carried out via cell culture studies using a ‘model electrically responsive cell line’, atrial muscle cells derived from the AT-1 mouse cardiomyocyte tumour lineage (HL-1 cells), to correlate any changes in bioactivity to those of the added attributes. In all characterisation cases, pure PCL was employed as the comparator scaffold (the experimental control).

The aim of this study was to showcase the use of a simple, rapid, and most importantly, a solvent-free FDM 3D printing regime to add the attributes of hydrophilicity and electroactivity to PCL to enhance its ability to support electrically responsive cells. It is hoped that this study will be a stepping stone towards the creation of electrically responsive tissue with increasing native-like structure and function for use in the much-needed tissue models to study physiological events and diseases and to test new interventions.

## 2. Material and Methods

### 2.1. Scaffold Preparation

An Allevi 2 3D Bioplotter (Allevi Inc., Philadelphia, PA, USA), with hot melt extrusion capability in the range of 20–160 °C, was employed to 3D fabricate a set of polycaprolactone scaffolds (PCL, MW 50,000, Polysciences Inc., Warrington, PA, USA) with increasing concentration of graphene (G) nanoplatelets (2–10 nm, ACS Material LLC, Pasadena, CA, USA) to include 0.75, 1.5, 3, and 6% (*w*/*w*) G, henceforth referred to as PCL+0.75G, PCL+1.5G, PCL+3G, and PCL+6G, respectively. The predetermined design of a ‘closed’ circular scaffold design, 12 mm in diameter with 0.75 mm height, was created using Computer Aided Design Software, Solid Edge 2020 (Siemens Corporation, Washington, D.C., USA) and uploaded as a .stl file to the bioplotter, where each scaffold composition was fabricated at 100 °C and 100 PSI, via a 27-gauge nozzle, at 0.1 mm layer thickness and 1 mm/s speed. In their as-received formats, PCL and G are solid state powders. PCL is white/off-white in colour and G is grey/black in colour. Each scaffold composition (0.75–6 G) was prepared by the simple addition of the relevant amounts of each powder to create PCL with 0, 0.75, 1.5, 3, and 6% (*w*/*w*) G. The powders were thoroughly mixed using a roller mixer prior to adding to the extruder barrel (a thermostable full metal barrel) with an attached extruder nozzle (thermostable full metal nozzle) and given 30 min to heat up to 100 °C and ‘melt’ prior to extrusion (the melting point of PCL is 62 °C [13]). The geometry of the grid was set at an infill distance of 0.335 mm to match that of the nozzle’s inner diameter to create a ‘closed’ structure (as solid as possible with no pores included in the design). In this study, the closed structure was chosen over that of one with predesigned 3D geometry (with appropriately sized pores to support cardiomyocytes) to ensure that material composition was fully investigated first (with a second follow-on investigation into predesigned 3D geometry anticipated).

Scaffold types were fabricated in turn, starting with PCL and ending with the highest concentration of PCL+G system (PCL+6G), with each extruded part (scaffold) taking 48 min to complete. To maximise successful fabrication, double-sided adhesive tape was added to the top and bottom of the glass microscope slide to adhere the slide to the build platform and the extruded scaffold to the slide.

### 2.2. Field Emission Scanning Electron Microscopy (FESEM)

FESEM (SU5000, Hitachi High-Tech Europe GmbH, Krefeld, Germany) was employed to study (i) the upper surface of the scaffolds before cell-seeding and (ii) cell morphology of adhered cells after cell-seeding (cell-seeding and FESEM of cell-seeded scaffolds are described in Section 2.7.1 and Section 2.7.2, respectively). An ultra-thin conductive palladium/gold layer (~18 nm) was deposited onto all scaffolds using an Emitech K500X coating system (Quorum Technologies, Lewes, UK) operating at 25 mA for 150 s. SEM images were then acquired for scaffolds at an acceleration voltage of 5 kV, under a low vacuum of 60 Pa combined with the backscattered electron detector, using a nominal spot size of 30 nm.

### 2.3. Raman Spectroscopy

Raman spectroscopy (Renishaw inVia™ Qontor^®^ Confocal Raman Microscope, Renishaw Ltd., Gloucestershire, UK) was employed to (i) confirm that G was exposed on the upper surface of the G-loaded scaffolds using a Raman ‘point and shoot’ method and (ii) observe the distribution of exposed G on the upper surface of the G-loaded scaffolds using a Raman ‘mapping’ method.

For Raman point and shoot, three random locations on the upper surface of each scaffold type were analysed in triplicate. Prior to commencing measurements, the Raman system was calibrated using an internal silicon reference to 520 cm^−1^. In acquisition mode, the laser was operated at 10% power (equal to 5 mW) and focused through a x20 objective over an extended wavenumber scan, 100–3500 cm^−1^, with 10 s integration time. Averaged spectra were data-processed by cosmic ray removal, if applicable, and baseline subtraction to remove any spectral artefacts. Averaged and data-processed Raman data were plotted and processed using Microsoft Excel^®^ (Microsoft Corporation, Redmond, WA, USA).

For Raman mapping, one random area (of specific surface area) at an approximate centre point on each scaffold type’s upper surface was analysed. Prior to commencing measurements, the Raman system was calibrated using an internal silicon reference to 520 cm^−1^. In acquisition mode, the laser was operated at 10% power (equal to 5 mW) and focused through a 5× objective over an extended wavenumber scan, 200–3500 cm^−1^, with 10 s integration time. On a square map, 400 µm in length and width, “Raman Intensity at Point” at 1577 cm^−1^, indicative of the G band in graphene, was measured every 10 µm steps. Spectra (1681 in total) were collected per map per scaffold type. Spectra were subjected to up to three data processing steps, cosmic ray removal, if applicable, baseline subtraction, and normalisation (by Raman intensity range using ‘1’ as the upper range and ‘0’ as the lower range), prior to generating false-colour black/green maps. Where increasing Raman intensity of 1577 cm^−1^ was detected, the map colour changed from ‘black’ colour to increasing ‘green’ colour (with increasing concentration of G).

### 2.4. X-ray Photoelectron Spectroscopy (XPS)

X-ray Photoelectron Spectroscopy (XPS) analyses were performed using an Axis Ultra DLD Spectrometer (Kratos Analytical Ltd., Manchester, UK). Spectra were analysed using monochromated Al K_α_ X-rays (hv = 1486.6 eV), operating at 10 mA and 15 kV. Wide energy survey scans were obtained from three random locations on each scaffold/sample at a 160 eV pass energy, with high-resolution scans of the C1s and O1s regions collected at a 40 eV pass energy. A charge neutraliser system was employed, operating at filament current of 1.95 A and charge balance of 3.3 V. In addition to the scaffolds, as-received G nanoplatelets (GNP) and PCL were XPS-analysed.

### 2.5. Electrochemical Impedance Spectroscopy (EIS)

To determine any electroactive effect from adding G to PCL, a Bio-Logic Potentiostat Galvanostat SP-200 (Biologic, Seyssinet-Pariset, France) was employed to generate EIS measurements on the scaffolds. Data were collected at room temperature in the Potentio EIS mode with a 3-electrode cell configuration, with each scaffold type acting as a working electrode. Cardiac cell specific medium (see Section 2.7) acted as the electrolyte solution. Prior to data collection, the working electrodes were submerged in electrolyte solution for 48 h at 2–8 °C to improve their wetting behaviour. Data were performed at the open circuit potential (OCP), with the application of an alternating current sinusoidal perturbation voltage of 10 mV (root mean square) at a frequency variation of 1 Hz to 1 MHz.

### 2.6. Water Contact Angle Measurement

The angle produced where a water/air interface met each scaffold was measured using a Goniometer and its associated software (CAM 200, KSV Instruments Ltd., Espoo, Finland) to determine surface and interface tensions on each scaffold type and their potential to support cells. A static method with a 5 µL water droplet as the liquid phase was used on each scaffold’s upper surface and the contact angle derived from curve-fitting using the Young/Laplace equation. Each contact angle measurement was taken on a random location on each scaffold’s upper surface and repeated on a new random location up to twelve times.

### 2.7. Cell Culture

Prior to biological characterisation, the scaffolds were exposed to germicidal ultra-violet (UV) light at wavelength 254 nm within the Class 2 Biosafety Cabinet (Bioquell UK Ltd., Andover, UK) to remove any microbial load; each outward-facing scaffold surface was subjected to 30 min of UV light before transfer to a sterile 12-well tissue culture plastic plate until the in vitro cell methods were performed.

Atrial muscle cells derived from the AT-1 mouse cardiomyocyte tumour lineage (HL-1 cells, gifted by Dublin City University, DCU, ROI) were employed as a ‘model electrically responsive cell line’ given their ability to continuously divide, spontaneously contract, and maintain a differentiated cardiac phenotype as well as their close similarity to primary cardiomyocytes [14,15]. 

In general, cells were cultured as per the recommendation of Claycomb et al. [14] in 0.02% gelatin/5 µg/mL fibronectin-coated tissue culture plasticware in Claycomb medium supplemented with 10% foetal bovine serum (FBS), 0.1 mM norepinephrine, 2 mM L-glutamine, and 100 U/mL:100 µg/mL penicillin/streptomycin, henceforth referred to as ‘medium’ and placed in a humidified incubator at 37 °C with 5% CO_2_ (standard incubation conditions). Cells were maintained at below 70% confluency and passaged every three days using 0.05% trypsin with 0.02% ethylenediamine tetra-acetic acid in sodium (EDTA-Na) followed by soybean trypsin inhibitor type I-S, as per the manufacturer’s protocol. Where possible, cells were monitored using an inverted microscope (Nikon Eclipse TS100, Nikon, Amsterdam, The Netherlands). All reagents were from Sigma-Aldrich (Merck, Dorset, UK).

#### 2.7.1. Cell-Seeding

At day 0, cells at passage number 27 had their concentration determined using an automated cell counter (TC20, Bio-Rad Laboratories Ltd., Hertfordshire, UK), as per the manufacturer’s protocol. Prior to cell-seeding, sterile scaffolds were coated with 0.02% gelatin/5 µg/mL fibronectin; then, 75 µL was pipetted onto the centre of each scaffold within sterile 12-well plates and incubated for 2 h under standard incubation conditions. Cell suspensions were standardised to 6.67 × 10^5^ cells per ml, and 75 µL of this suspension (50,000 cells per scaffold) pipetted onto the centre of each scaffold. Cell-seeded scaffolds were incubated for 2 h under standard conditions to maximise cell adhesion, after which 1925 µL of medium was added. Plates were incubated under standard conditions until each experimental timepoint (days 1, 2, and 3) was reached, with medium replenished daily. Tissue culture plastic (TCP) controls were included and treated in the same manner as those of the test scaffolds described above. Negative TCP controls consisted of 75 µL of medium only (medium, no cells or scaffold), and positive TCP controls consisted of 75 µL of cell suspension only (cells and medium, no scaffold). Negative TCP controls were used to demonstrate the maintenance of aseptic conditions, and positive TCP controls to demonstrate the merit of the cell line.

#### 2.7.2. Scanning Electron Microscopy of Adhered Cells

FESEM (SU5000, Hitachi High-Tech Europe GmbH, Krefeld, Germany) was employed to study (i) the upper surface of the scaffolds before cell-seeding (see Section 2.2) and (ii) cell morphology of adhered cells after cell-seeding. At day 3, the morphology of any adhered cells was observed. Medium was removed from their wells, wells washed twice with 0.01 M phosphate buffered saline (PBS), and scaffolds chemically fixed with 2.5% glutaraldehyde in water for 45 min at room temperature followed by two washes with 0.01 M PBS and further followed by two washes of purified water. Scaffolds were gradually dehydrated using an alcohol series of increasing ethanol concentration, 25, 50, 75, 90, and 100% ethanol, for 8 min at room temperature followed by a 1:1 ratio of 100% ethanol and 100% hexamethyldisilizane (HMDS) for 8 min. Scaffolds were chemically dried overnight at room temperature with 100% HMDS and then palladium/gold-coated (see Section 2.2).

#### 2.7.3. 4′,6-Diamidino-2-phenylindole (DAPI)-Staining of Adhered Cells

An indication of cell numbers on exposure to the scaffolds was determined at day 3 by staining the nuclei of any adhered cells. Medium was aspirated from wells of test scaffolds and controls. Wells were washed twice with 0.01 M PBS and cells chemically fixed on incubation with 4% paraformaldehyde in 0.01 M PBS for 8 min at room temperature. Wells were washed twice with 0.01 M PBS before staining cells with 300 nM 4′,6-diamidino-2-phenylindole (DAPI) in 0.01 PBS for 10 min at room temperature. Wells were washed twice with 0.01 M PBS followed by two further washes with purified water before immediate visualisation using a wide-field fluorescent microscope and its associated software (Zeiss Axio Imager, Zeiss GmbH, Aalen, Germany). At least 3 random areas per DAPI-stained scaffold were imaged using Colibri 7 LED illumination at an excitation wavelength of 405 nm for 820 milliseconds. Images were captured at 1024 × 1024 pixels of resolution.

#### 2.7.4. In Vitro Cell Method to Measure Cell Viability of Adhered Cells

At days 1, 2, and 3, an indication of cell viability on exposure to the scaffolds was determined using AlamarBlue™ HS Cell Viability Reagent (ThermoFisher Scientific, Waltham, MA, USA). Medium was removed from wells containing test substrates and controls. Viability reagent was added at a working concentration of 1:9 ratio mixture of viability reagent to medium within wells and incubated under standard conditions for 2 h in the dark. Following thorough mixing, aliquots of the solution (3 × 100 µL per well, n = 9 per scaffold type) were transferred to a black 96-well microplate, and absolute fluorescence intensity (arbitrary units) measured using a Tecan Spark Spectrophotometer (Tecan Group Ltd., Männedorf, Switzerland) at an excitation wavelength of 560 nm and an emission wavelength of 590 nm. Each scaffold type was tested in triplicate (n = 9 per scaffold type). If used correctly, viability reagent is non-toxic to cells; hence, wells containing test substrates and controls were replenished with 2 mL of medium and returned to standard incubation conditions until the next timepoint.

### 2.8. Statistical Analyses

Statistical analyses of the results from contact angle measurement and in vitro cell methods (n = 3 minimum, n = 12 maximum) were performed using GraphPad Prism version 9.3.1 for Windows (GraphPad Software, San Diego, CA, USA). A one-way analysis of variance (ANOVA) was applied to test for statistically significant differences at each timepoint, with a value of *p* < 0.05 considered statistically significant. Any significant differences in the results for pure PCL, the experimental control, and those on the four types of G-loaded scaffolds (0.75, 1.5, 3, and 6 G) were determined using Dunnett’s multiple comparisons test, with a value of *p* < 0.05 taken as statistically significant.

## 3. Results

### 3.1. FESEM

**Figure 1:**
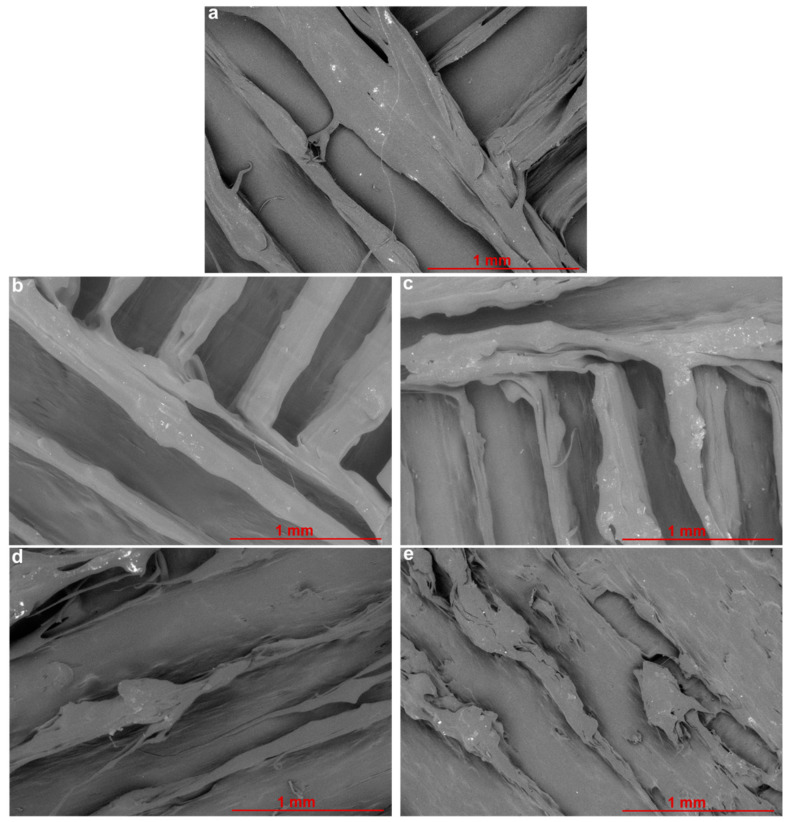
FESEM images from one random location on the upper surface of 3D-fabricated scaffolds, (**a**) PCL, (**b**) PCL+0.75G, (**c**) PCL+1.5G, (**d**) PCL+3G, and (**e**) PCL+6G, at 50× magnification. SEM images were acquired at an acceleration voltage of 5 kV, under a low vacuum of 60 Pa combined with the backscattered electron detector, using a nominal spot size of 30 nm. Scale bar represents a 1 mm distance within the image.

Qualitative information on the scaffolds’ upper surface topography is provided by FESEM images for PCL and PCL+G scaffolds created by fused deposition modelling (FDM), Figure 1, with the surface region of hot-melt-extruded filaments shown for both PCL (Figure 1a) and each of the PCL+G (Figure 1b–e) systems. It is noted that the layer-by-layer printing process is observed in these images, with the uppermost layer perpendicular to the underlying layer. Additionally, the predefined design of creating a ‘closed’ structure (as solid as possible with no pores) by setting the infill distance at 0.335 mm to match that of the nozzle’s inner diameter is also evident in the images; Figure 1a–e demonstrate that the horizontally deposited parallel filaments are in contact with one another.

Fabrication of the scaffolds by 3D hot extrusion printing became increasingly more difficult as the G concentration increased; on comparing the pure PCL scaffold (Figure 1a) to the G-loaded scaffolds (Figure 1b–e), the extruded filaments lose uniformity, displaying more fragmented structure and giving a somewhat uneven and ‘ragged’ appearance as the G concentration increases. The most uneven topography/roughness is visible for PCL+3G and PCL+6G scaffolds (Figure 1d,e, respectively).

### 3.2. Raman Plots

**Figure 2:**
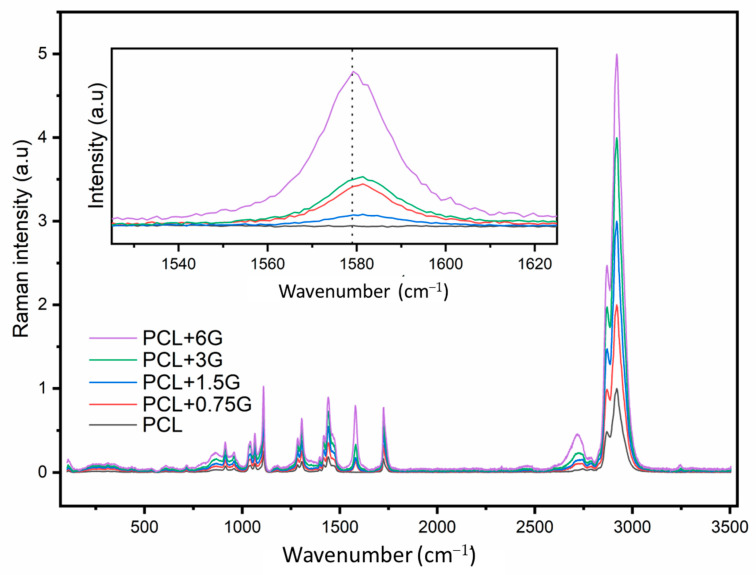
Raman spectra from one random location on the upper surface of 3D-fabricated scaffolds: main image represents PCL, PCL+0.75G, PCL+1.5G, PCL+3G, and PCL+6G (see colour key), and inserted image with zoomed-in region (1525–1625 cm^−1^) demonstrates the varying Raman intensity at 1577 cm^−1^ for PCL, PCL+0.75G, PCL+1.5G, PCL+3G, and PCL+6G (see colour key). Three random locations on the upper surface of each scaffold type were analysed in triplicate using a 532 nm laser at 10% power (5 mW) and focused through a x20 objective over an extended wavenumber scan, 100–3500 cm^−1^, with 10 s integration time. Averaged spectra were data-processed by cosmic ray removal, if applicable, and baseline subtraction. The x-axis is Wavenumber in cm^−1^, and the y-axis is Raman intensity in arbitrary units (a.u).

Raman spectroscopy confirmed that G is exposed on the upper surface of the G-loaded scaffolds, as opposed to being encapsulated in PCL (a possibility due to the melting process prior to extrusion). This is confirmed by a Raman peak at 1577 cm^−1^, which is indicative of the G band associated with graphene, in the spectra for G-loaded PCL scaffolds (PCL+0.75G, PCL+1.5G, PCL+3G, and PCL+6G), as shown in Figure 2, with the same figure demonstrating the absence of said peak in the spectrum for the pure PCL scaffold, as expected. The inserted zoomed-in image in Figure 2 demonstrates the varying intensity of the Raman peak at 1577 cm^−1^ across the G gradient, that, in general, increases across the gradient (0.75–6% G). There was a marked increase in intensity from the PCL+3G to PCL+6G scaffold. All spectra demonstrate highly similar spectral profiles with eight common Raman peaks for PCL detected at ~909, 1108, 1307, 1438, 1725, 2748, 2871, and 2921 cm^−1^ (Figure 2).

**Figure 3:**
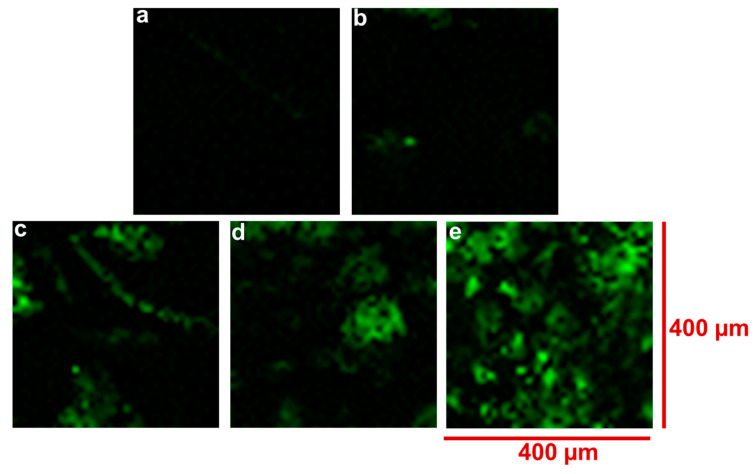
False-colour Raman maps for 3D-fabricated scaffolds, (**a**) PCL, (**b**) PCL+0.75G, (**c**) PCL+1.5G, (**d**) PCL+3G, and (**e**) PCL+6G. One random area (400 × 400 µm^2^) on each scaffold type’s upper surface was analysed. The 532 nm laser was operated at 10% power (5 mW) and focused through a 5× objective over an extended wavenumber scan, 200–3500 cm^−1^, with 10 s integration time. On a square map, 400 µm in length and width, “Raman Intensity at Point” at 1577 cm^−1^ was measured every 10 µm steps. Spectra (1681 in total) were collected per map per scaffold type and subjected to up to three data processing steps, cosmic ray removal, if applicable, baseline subtraction, and normalisation, prior to generating false-colour black/green maps. On increasing Raman intensity of 1577 cm^−1^ (indicative of graphene), the map colour changed from ‘black’ colour to increasing ‘green’ colour.

Raman spectroscopy was also employed to give qualitative information on the distribution of G on the upper surface of the scaffolds by mapping the intensity of the Raman peak at 1577 cm^−1^ across a specific surface area (400 µm^2^) of each scaffold type. The intensity of G detected is represented by the presence of ‘green’ colouration in the Raman maps presented in Figure 3. As expected, no green colouration was detected for the pure PCL scaffold (Figure 3a), with increasing intensity of ‘green’ colouration seen for the G-loaded PCL scaffolds (Figure 3b–e), demonstrating the increasing concentration and distribution of G within the PCL-based filaments of the scaffolds. The maps shown in Figure 3b–e appear to indicate an even distribution of ‘green’ colouration due to G.

### 3.3. XPS

**Figure 4:**
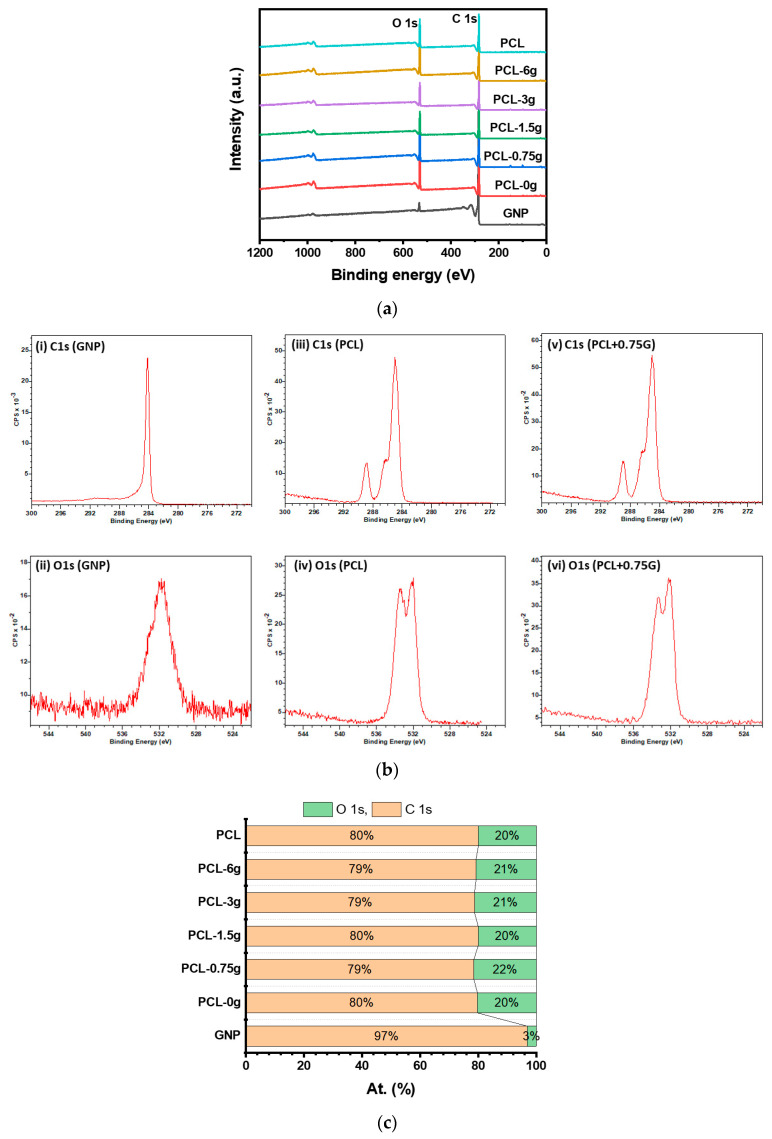
(**a**) Wide Energy Survey Scans (WESS) for the as-received G nanoplatelets (GNP), 3D-fabricated scaffolds (PCL, PCL+0.75G, PCL+1.5G, PCL+3G, and PCL+6G) and as-received PCL (in ascending order). X-ray spectra were collected using monochromated Al K_α_ X-rays (hv = 1486.6 eV), operating at 10 mA and 15 kV, with WESS obtained from three random locations on each scaffold at a 160 eV pass energy. A charge neutraliser system was employed, operating at filament current of 1.95 A and charge balance of 3.3 V. The x-axis is Binding energy (eV) and the y-axis is XPS intensity in arbitrary units (a.u.). (**b**) High resolution scans for GNP ((i) C1s, (ii) O1s), PCL ((iii) C1s, (iv) O1s), and PCL+0.75G ((v) C1s, (vi) O1s). X-ray spectra were collected using monochromated Al K_α_ X-rays (hv = 1486.6 eV), operating at 10 mA and 15 kV, with high resolution scans of the C1s and O1s regions obtained from three random locations on each scaffold at a 40 eV pass energy. A charge neutraliser system was employed, operating at filament current of 1.95 A and charge balance of 3.3 V. The x-axis is Binding energy (eV) and the y-axis is Counts per second (CPS). (**c**) Percentage atomic concentration (At%) data for elemental orbitals O1s and C1s derived from the XPS analysis of one random location on the upper surface of as-received G nanoplatelets (GNP), 3D-fabricated scaffolds (PCL, PCL+0.75G, PCL+1.5G, PCL+3G, and PCL+6G), and as-received PCL (in ascending order). The x-axis is At %, and the y-axis is Sample type.

XPS survey scans (0–1200 eV) from one random location on the upper surface (<10 nm) for the as-received G nanoplatelets (GNP), as-received PCL powder (PCL+0G), and 3D-fabricated scaffolds are shown in ascending order in Figure 4a. Only peaks assigned to a C1s at ~285–289 eV and O1s at ~533.7 eV photoemission are present in each case. High-resolution scans of the C1s and O1s regions for the as-received G nanoplatelets (GNP), the as-received PCL powder (PCL+0G), and the 3D-fabricated PCL scaffold with 0.75% (*w*/*w*) GNP (PCL+0.75G) are shown in Figure 4b. The percentage atomic concentration (At%) for the C1s and O1s contributions detected in each sample type is presented in Figure 4c. The At% C1s and O1s contributions for the as-received G were 97% and 3%, respectively, while those for as-received PCL and 3D fabricated scaffolds were all in the ranges 79–80% C1s and 21–20% O1s.

### 3.4. EIS

**Figure 5:**
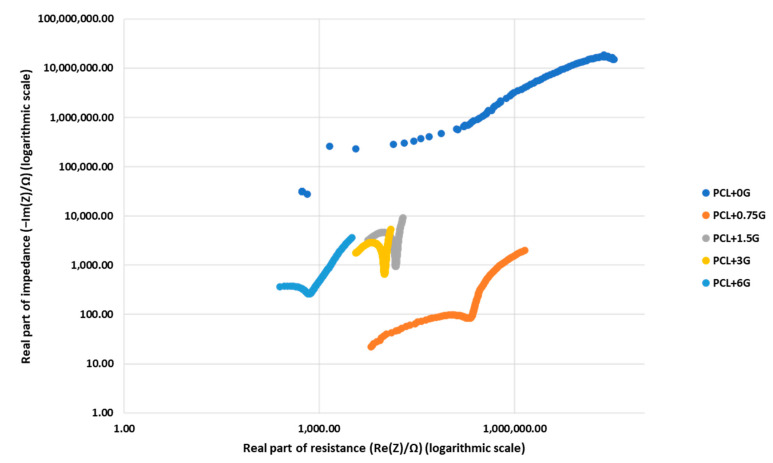
Nyquist plots for all 3D-fabricated scaffolds, PCL, PCL+0.75G, PCL+1.5G, PCL+3G, and PCL+6G (see key), generated from EIS measurements with a 3-electrode cell configuration, with each scaffold type acting as a working electrode. Cardiac cell specific medium acted as the electrolyte solution, and prior to data collection, the working electrodes were submerged in electrolyte solution for 48 hours at 2−8 °C. Data were performed at the open circuit potential, with the application of an alternating current sinusoidal perturbation voltage of 10 mV (root mean square) at a frequency variation of 1 Hz to 1 MHz. The x-axis is Real part of resistance in Ω (logarithmic scale), and the y-axis is Real part of impedance in Ω (logarithmic scale).

**Figure 6:**
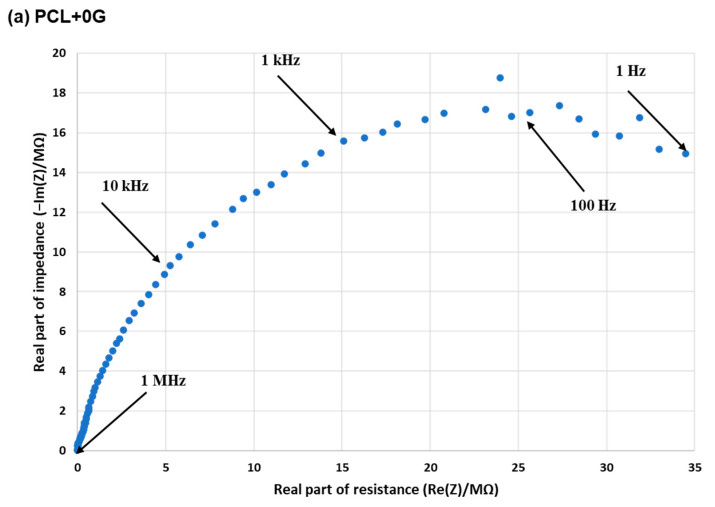

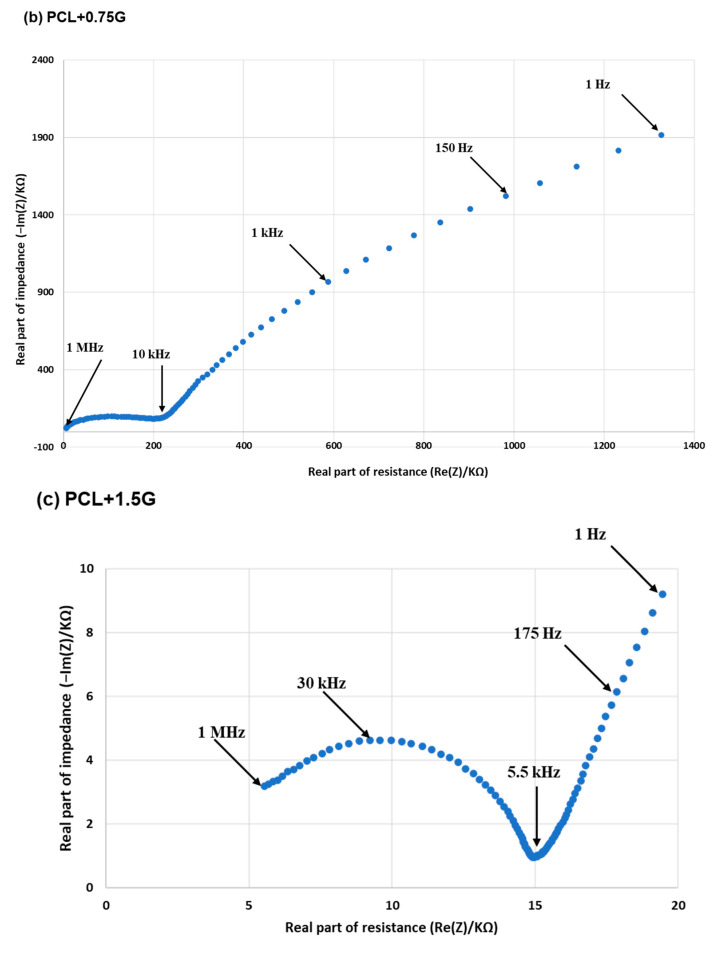

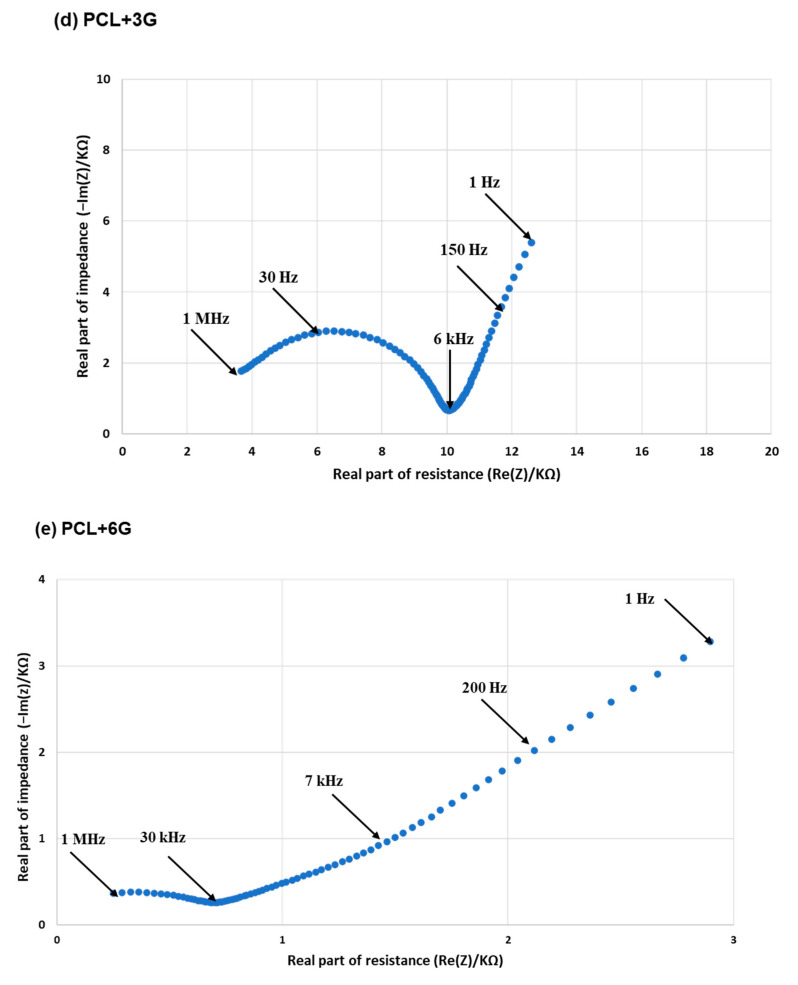
Nyquist plots for 3D-fabricated scaffolds, (**a**) PCL, (**b**) PCL+0.75G, (**c**) PCL+1.5G, (**d**) PCL+3G, and (**e**) PCL+6G, generated from EIS measurements with a 3-electrode cell configuration, with each scaffold type acting as a working electrode. Cardiac cell specific medium acted as the electrolyte solution, and prior to data collection, the working electrodes were submerged in electrolyte solution for 48 h at 2–8 °C. Data were performed at the open circuit potential, with the application of an alternating current sinusoidal perturbation voltage of 10 mV (root mean square) at a frequency variation of 1 Hz to 1 MHz. The x-axis is Real part of resistance (in MΩ for (**a**) and kΩ for (**b**–**e**)), and the y-axis is Real part of impedance (in MΩ for (**a**) and kΩ for (**b**–**e**)). Some changing frequencies are highlighted.

Nyquist plots representing the real and the imaginary parts of impedance measurements for each scaffold type are provided in Figure 5 and Figure 6 as a measure of their electroconductivity. Figure 5 shows all EIS data for the five scaffolds on one Nyquist plot to aid in the comparison of impedance changes, while Figure 6 shows five Nyquist plots, one per scaffold, to aid in EIS data clarity per scaffold. The graphical data plots can be divided into two parts, with the first being the high-frequency region, which exhibits a semi-circular arc where the bending implies the operation of a charge transfer mechanism. The diameter of the semi-circle in the Nyquist plot allows for an estimation of the magnitude of the charge transfer resistance of the working electrode (scaffold). In the low-frequency range, a linear region can be observed, which implies the occurrence of a diffusion mechanism [16]. Figure 6a demonstrates a semi-circular plot with a large real and imaginary impedance (in MΩ) for the experimental control, the pure PCL scaffold, with the broad diameter signifying a hindering of any swift charge transfer and indicating the expected poor electroconductivity. Figure 6b–e demonstrates that the impedance of PCL is reduced significantly by the presence of G and decreases with increasing G concentration, indicating an improvement in electroconductivity from PCL+0.75G to PCL+6G. For PCL+0.75G, the diameter of the semi-circle (~207 kΩ) is reduced by almost 2 orders of magnitude compared with that for the pure PCL scaffold (Figure 6a,b, respectively). This significant reduction of both the real and imaginary parts of the impedance plot continued on increasing G concentration wherein the diameter of the semi-circles decreased from ~9, 6, and 0.5 kΩ for PCL+1.5G, PCL+3G, and PCL+6G, respectively, thereby indicating an increase in scaffold electroconductivity on increasing G concentration.

### 3.5. Water Contact Angle Measurement

**Figure 7:**
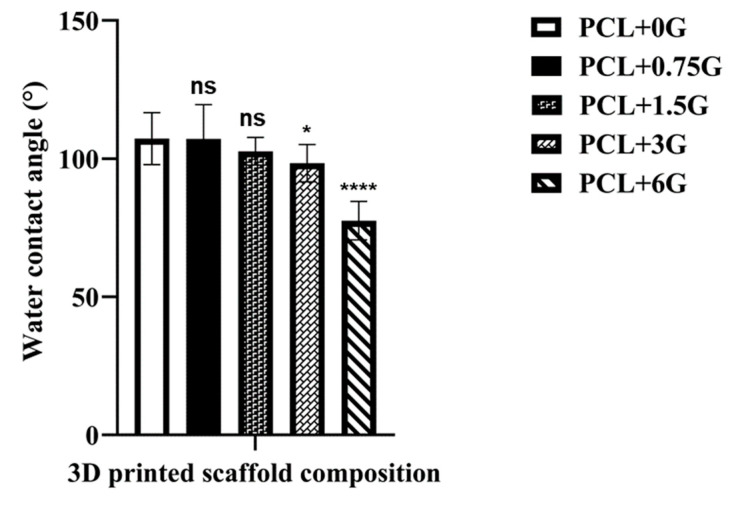
Mean water contact angle (°) values for 3D-fabricated scaffolds, PCL, PCL+0.75G, PCL+1.5G, PCL+3G, and PCL+6G (see key). A 5 µL water droplet was used on each scaffold’s upper surface, and the contact angle derived from curve-fitting using the Young/Laplace equation. Each contact angle measurement was taken on a random location on each scaffold’s upper surface and repeated on a new random location up to twelve times. Errors bars represent mean +/− standard deviation. Statistical significance in comparison with pure PCL, as determined by Dunnett’s multiple comparisons test, is represented by either not significant (ns), where *p* > 0.05, or significant, where *p* < 0.05. Increasing significance is represented by an increasing number of asterisks (* *p* < 0.05 and **** *p* < 0.0001). The x-axis is 3D printed scaffold composition, and the y-axis is Water contact angle (°).

Mean water contact angles for the core sample set of interest here are shown in Figure 7. These data show a decrease in contact angle with increasing G concentration. Scaffolds containing the highest G concentration (PCL+3G and PCL+6G) had statistically significant lower contact angles (98.33 ± 6.75 and 77.56 ± 6.75°, respectively) compared with that for pure PCL (107.22 ± 9.39°).

### 3.6. Scanning Electron Microscopy of Adhered Cells

**Figure 8:**
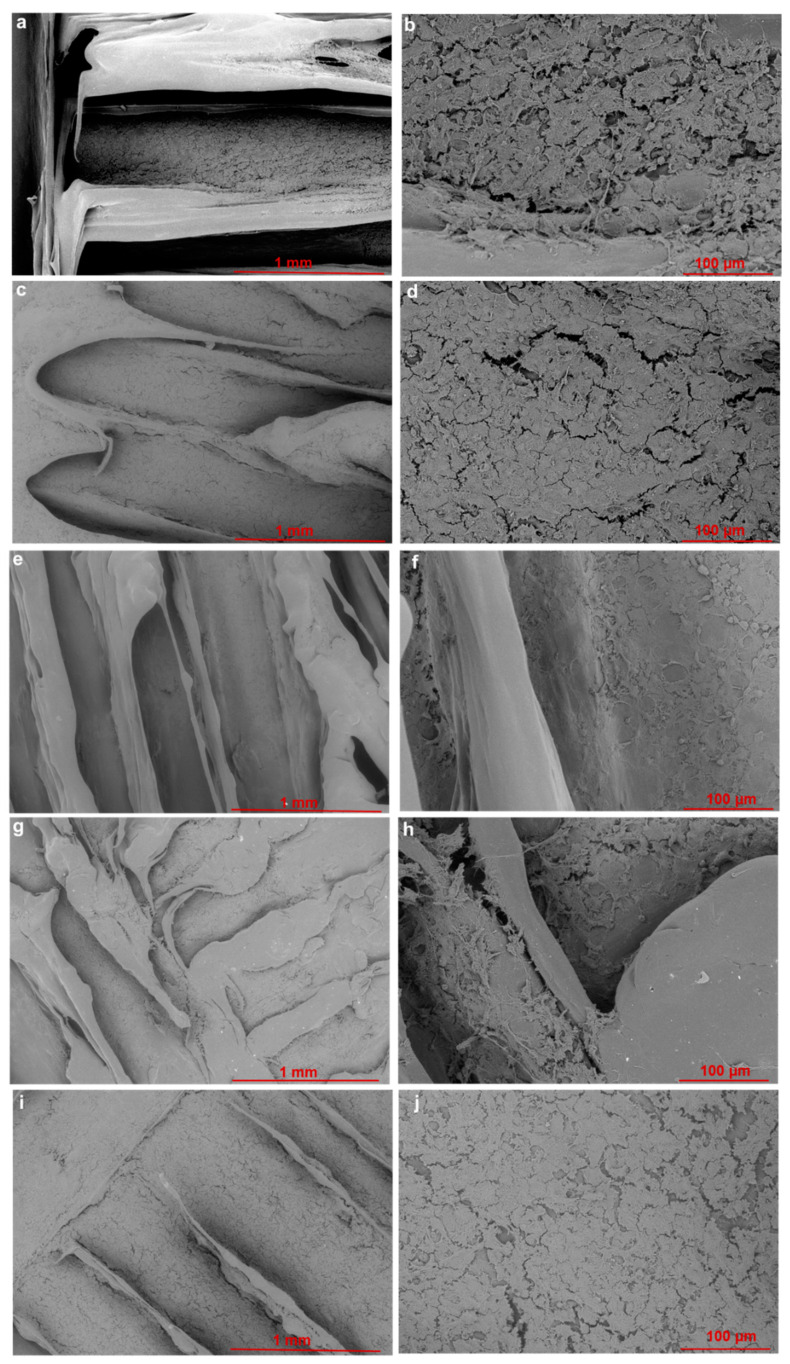
FESEM images taken from one random location for HL-1 cells exposed to PCL and PCL+G scaffolds cultured under standard conditions for 3 days at (i) 50× maginification: (**a**) PCL, (**c**) PCL+0.75G, (**e**) PCL+1.5G, (**g**) PCL+3G, and (**i**) PCL+6G, and at (ii) 300× magnification: (**b**) PCL, (**d**) PCL+0.75G, (**f**) PCL+1.5G, (**h**) PCL+3G, and (**j**) PCL+6G. SEM images were acquired at an acceleration voltage of 5 kV, under a low vacuum of 60 Pa combined with the backscattered electron detector, using a nominal spot size of 30 nm. Scale bar represents a 1 mm distance within the image (**a**,**c**,**e**,**g**,**i**) and 100 µm distance within the images (**b**,**d**,**f**,**h**,**j**).

Qualitative information on adhered cell morphology is provided by images for AT-1 mouse atrial cardiomyocyte tumour lineage (HL-1) cells on the PCL and PCL+G scaffolds at day 3, Figure 8. It should be noted that during FESEM image acquisition, cells were easily found on all scaffolds and were always visible during x and y movements. All scaffolds exhibited cell coverage across the focal view of each image at 50× magnification (Figure 8a,c,e,g,i). Cells are adhered to the extruded filaments on both the upper and underlying filaments. At 300× magnification, cell clusters of relatively ‘flat’ morphology are clearly identifiable, along with the lamellipodia of cell–cell adhesions (Figure 8b,d,f,h,j). The underlying scaffold surface is visible between the areas of cell clustering (Figure 8b,d,h).

**Figure 9:**
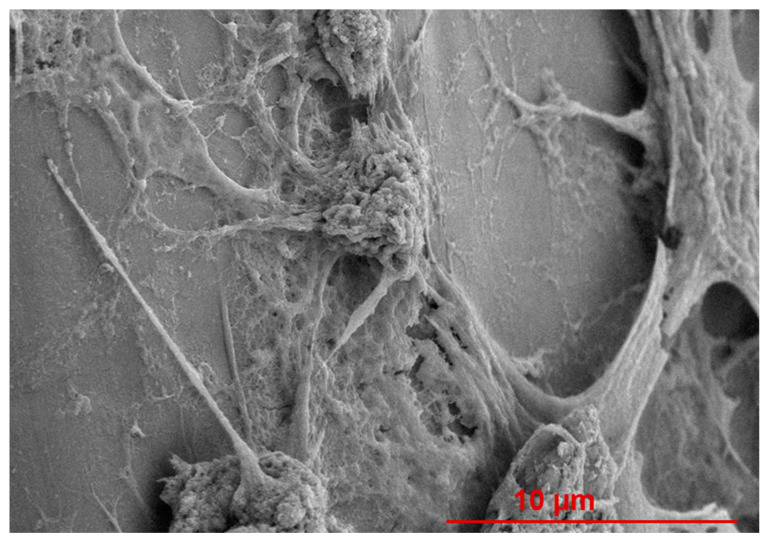
FESEM image at 5000× magnification taken from one random location for HL-1 cells exposed to a PCL+1.5G scaffold cultured under standard conditions for 3 days. SEM image was acquired at an acceleration voltage of 5 kV, under a low vacuum of 60 Pa combined with the backscattered electron detector, using a nominal spot size of 30 nm. Scale bar represents a 10 µm distance within the image.

Figure 9 provides a 5000×-magnified view of a cell-seeded PCL+1.5G scaffold. Some cell features are clearly visible: cell body, filopodia, and lamellipodia. Cell bodies with both ‘flat’ and ‘raised’ conformation are visible along with epithelial-like cell morphology, cell-stretching, cell-to-cell adhesions, and cell-to-scaffold adhesions. Dimensionally, individual cells are in the range of ~5–10 µm.

### 3.7. DAPI-Staining of Adhered Cells

**Figure 10:**
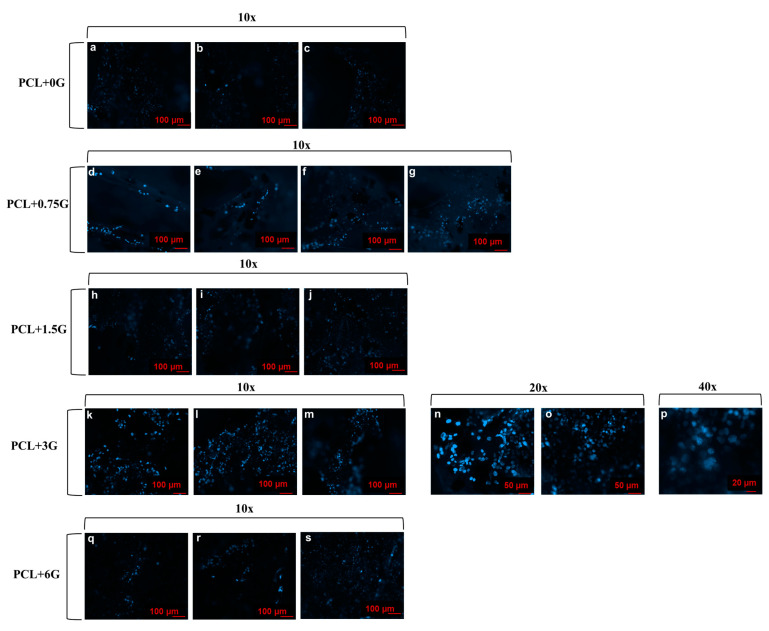
Wide-field fluorescence images for nuclei-stained HL-1 cells from random locations on PCL+0G (experimental control), PCL+0.75G, PCL+1.5G, PCL+3G, and PCL+6G scaffolds cultured under standard conditions for 3 days and acquired at several magnifications (see key). At least 3 random areas per DAPI-stained scaffold were imaged using Colibri 7 LED illumination at an excitation wavelength of 405 nm for 820 milliseconds. Images were captured at 1024 × 1024 pixels of resolution. Scale bar represents a 100 µm distance within images (**a**–**m**) and (**q**–**s**), a 50 µm distance within the images (**n,o**), and a 20 µm distance within image (**p**).

Qualitative information on the number of cells which adhered to PCL and PCL+G scaffolds after 3 days in culture is presented as fluorescent micrographs of stained nuclei of adhered cells taken at several magnifications in Figure 10. As is the case for the FESEM images, all scaffolds exhibit good nuclei coverage present across the focal view of each image. Nuclei are present on the curvature of the extruded filaments, which accounts for the slight loss of focus within each micrograph, with only some nuclei fully in focus within each image.

### 3.8. In Vitro Cell Method to Measure Cell Viability of Adhered Cells

**Figure 11:**
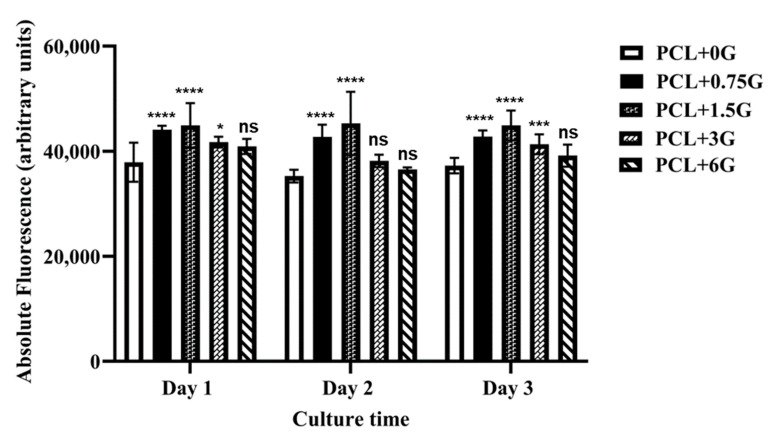
Mean Alamar Blue™ HS Cell Viability Assay data from HL-1 cells exposed to 3D-fabricated scaffolds, PCL, PCL+0.75G, PCL+1.5G, PCL+3G, and PCL+6G (see key) under standard culture conditions for 1, 2, and 3 days. Absolute fluorescence was measured at an excitation wavelength of 560 nm and an emission wavelength of 590 nm. Each scaffold type was tested in triplicate. Error bars represent standard deviation. Statistical significance in comparison with pure PCL, as determined by Dunnett’s multiple comparisons test, is represented by either not significant (ns), where *p* > 0.05, or significant, where *p* < 0.05. Increasing significance is represented by an increasing number of asterisks (* *p* < 0.05, *** *p* < 0.001, and **** *p* < 0.0001). The x-axis is Culture time (in days), and the y-axis is Absolute fluorescence in arbitrary units (a.u.).

Quantitative data on the ability of the 3D-fabricated scaffolds to support viable cells was obtained by using the Alamar Blue™ HS Cell Viability Reagent on cells seeded on each scaffold type at days 1, 2 and 3 in culture, with the results shown in Figure 11. These data demonstrate that cell viability, as determined by the absolute fluorescence from cells over a 3-day culture period, is in the range ~33,000–54,000 a.u. In general, the results for each scaffold type per timepoint remained relatively static as a function of the culture time. For example, the mean fluorescence intensities for cells exposed to PCL+0.75G scaffolds were 44,111.22 ± 682.53, 42,719.22 ± 2333.68, and 42,791.67 ± 1181.00 at days 1, 2, and 3, respectively. However, at all timepoints, cells cultured on all G-loaded scaffolds (PCL+0.75-PCL+6G) produced higher fluorescence intensity values than those exposed to the pure PCL scaffold. Overall, the PCL+0.75G and PCL+1.5G scaffolds demonstrated the highest degree of cell viability (*p* < 0.0001) compared with the pure PCL scaffold. The PCL+3G scaffold demonstrated statistically significant increases in viability at days 1 and 3 only (*p* < 0.05 and *p* < 0.001, respectively).

## 4. Discussion

In this work, 3D printing was employed to fabricate a series of PCL scaffolds with increasing G content (0.75–6% G) in an attempt to introduce attributes of hydrophilicity and electrical activity to promote scaffold bioactivity with electrically responsive cells. A hot melt extrusion 3D printing process was used to fabricate model scaffolds from PCL and each PCL+G material. The hydrophilicity, electroactivity, and bioactivity of the PCL and PCL+G scaffolds were assessed using a range of physico-chemical characterisation methods: FESEM, XPS, Raman spectroscopy, and water contact angle. Electrical characterisation was undertaken by EIS, and biological assessment methods employed included FESEM imaging of fixed cells, visualisation of fluorescently stained nuclei, and a cell viability assay.

In FDM 3D printing, deposition occurs via continuous hot melt extrusion of a filament that is ‘laid down’ in a ‘line-by-line’ and ‘layer-by-layer’ regime until the relevant part is manufactured. The size and shape of the extruded part is controlled by the predetermined design executed via a CAD-derived .stl file. Operation of the Allevi 2 Bioplotter used here is Internet-browser-based, which allows for remote fabrication from an appropriate on-line-enabled device. The materials used here to create scaffolds are PCL and G powders in appropriate amounts to create 0.75, 1.5, 3, and 6% (*w*/*w*) G mixtures. Thorough mixing is employed to increase the homogeneity within the extruder barrel and aid subsequent hot melt extrusion. This solvent-free scaffold preparation methodology was used to avoid the possibility of residue solvent in the 3D structure that might adversely affect subsequent in vitro cell response. Other published works, such as Hitscherich et al. and Ginestra, employed solvents (acetone and cyclopentatone, respectively) to create PCL+G mixtures which involved heating, sonication, and proficient stirring, i.e., solution-blending, to thoroughly disperse G within PCL at lower concentrations (0.005–0.05% and 1–2%) [9,12]. By necessity, the scaffolds required for this work employed higher G concentration (0.75–6%) to produce the required hydrophilicity and electrical conductivity properties and so, compensation for any deficiency in G dispersal at lower levels of G inclusion was not required. The use of a solvent-free printing regime is highly advantageous and much sought-after, eliminating any environmental concerns and allowing printing to occur in the open environment [17]. Additionally, the use of any solvent (or other additives) and ultrasonication or mechanical agitation can negatively impact the properties of G and G-derivatives [17].

On addition of increasing amounts of G to PCL, there was a colour change in the mixtures from white/off-white to grey/black, which then was also seen in the resulting 3D-fabricated scaffolds. FESEM imaging confirmed that the predetermined ‘closed’ structure design was reproduced for the pure PCL scaffold and, furthermore, was generally well-maintained for the lower G concentrations (0.75–3%) used here. However, the extruded filaments do lose uniformity and become ‘rougher’ compared with the pure PCL system as the amount of G is increased, with the most uneven topography/roughness visible for the PCL+3G and PCL+6G scaffolds. On increasing G concentration from 3% upwards, fabrication became more difficult, with excess material clinging to the nozzle tip and being dragged behind the nozzle, thus causing disruption to the evenness of the layer-by-layer deposition. Unlike pure PCL, which melted completely prior to extrusion, the PCL+G mixtures did not. Graphene has an extremely high melting temperature, with previous studies estimating it to be 4236.85 °C for freestanding G [18]. A study by Ganz et al., which employed exclusively reliable ab initio molecular dynamics calculations to study the initial stages of melting of freestanding G monolayers, found that melting only starts at 4726.85 °C [18]. Hence, it is to be it expected that loading a polymer that melts at a relatively low temperature (100 °C) with an additive that melts at a much higher temperature will affect the viscosity of the mixture within the extruder barrel, making the pure PCL extrusion parameters sub-optimal. Three-dimensional printing issues with G were expected by the authors; a recent review by Wu et al. highlighted the aggregation and overlaying issues with 3D printing G nanoflakes [17]. In this study, the presence of G may have required an increase in the barrel shear force during extrusion beyond the 100 PSI used for pure PCL and may also have benefitted from the 1 mm/s filament extrusion rate used for pure PCL fabrication being increased to negate some of the fabrication difficulties. However, such adjustments were not performed, but rather the same fabrication parameters were used for all scaffold types.

Another consideration that influenced the choice of melt-blending over solvent-blending here is the need for the G component not to be encapsulated by the PCL during scaffold fabrication so that it is available to interact with cells that are seeded thereon; polymer encapsulation of additives is a common phenomenon that can occur during polymer extrusion [19,20]. To check for this effect, the upper surface of the 3D-fabricated scaffolds was analysed by Raman spectroscopy using both Raman ‘point and shoot’ and mapping methods, using the key Raman peak for G at ~1577 cm^−1^ at a depth of analysis of ~0.7 µm, with a 532 nm laser employed. Caetano et al. employed similar Raman methods to successfully confirm the presence and distribution of G in their 3D-printed PCL+G scaffolds [7]. While there are other Raman peaks indicative of G, at 1340 and 2692 cm^−1^, corresponding to the D and 2D bands, respectively, the G band peak at ~1577 cm^−1^ is usually the most intense [7,21,22,23]. As expected, there was no Raman peak at ~1577 cm^−1^ detected in the spectrum for the pure PCL scaffold. A significant peak at 1577 cm^−1^ was detected in the Raman spectra for all four G-loaded PCL scaffolds (PCL+0.75G, PCL+1.5G, PCL+3G, and PCL+6G) in each of the three separate locations analysed on each scaffold surface. In general, the 1577 cm^−1^ peak increased in intensity on increasing G concentration (0.75–6% G, Figure 2), reflecting the additional analytical worth of Raman spectroscopy to inform on concentration changes. These results confirm that the melt-blending method used here did not cause polymer encapsulation of G on the surface of the hot-melt-extruded filaments. Further confirmation of this condition came from the Raman chemical maps generated for each scaffold over a larger surface area of 400 µm^2^ than that for the spectra. Not only was G confirmed to be present on the uppermost surface of each scaffold, it was found to be well-distributed over the 400 µm^2^ area. This was found to be the case for all the PCL+G scaffolds studied (0.75–6%), with the intensity of the detected Raman signal for G increasing in line with its concentration in the mixtures used to 3D print the scaffolds. The latter result is a valuable outcome given that typically homogeneous/uniform dispersion is difficult to achieve with melt-blending, and that is often the reason for choosing solvent blending [24,25]. Caetano et al. reported uniform distribution of G in their 3D-printed PCL+G scaffolds, but they, too, chose to melt-blend the PCL and G [7]. In addition to the Raman peaks indicative of G, eight other Raman peaks were commonly detected and, as expected, are assigned to vibrational modes for the PCL in the scaffolds: at ~909 (C-COO stretch), 1108 (C-C stretch), 1307 (CH_2_ twist), 1438 (CH_2_ bend), 1725 (C=O stretch), 2748, 2871, and 2921 cm^−1^ [26,27,28].

XPS analyses were performed on the uppermost surface (<10 nm) of the 3D-fabricated scaffolds in addition to as-received GNP (G) and as-received PCL. The results highlighted in Figure 4a,b clearly show the presence of the expected C1s and O1s peaks at ~285–289 eV and ~533.7 eV, respectively [24,25,26,27,28]. However, it is noticeable that the O1s peak intensity and subsequent At% is much lower for the as-received G sample (3%) when compared with the as-received PCL powder or the PCL and PCL+G scaffolds (typically 20–22%). The marked increase in XPS intensity of the O1s peak (and its At%) from the as-received G to the as-received PCL and 3D fabricated scaffolds is owing to the increasing addition of carbon–oxygen groups from the PCL matrix. The O/C ratios calculated from Figure 4c are all very similar for the as-received PCL and 3D-fabricated scaffolds, showing minimal variation among the samples. As such, the addition of G here to the surface of the PCL does not appear to change the chemistry of the PCL, certainly at the scaffold depth, interrogated by the XPS conditions employed here: ~5–10 nm. It was seen earlier that when a larger depth of analysis was interrogated by Raman spectroscopy, ~700 nm, G was indeed detected.

Evidence of electrical conductivity in the PCL+G scaffolds due to the inclusion of G is provided by EIS measurements. The corresponding Nyquist plots provide significant information on the changes in the conductivity as the amount of G (0.75–6%) is increased in the PCL+G scaffolds used as working electrodes in the EIS set-up. Specifically, the intensity of the impedance component and the diameter of the semi-circle in the high-frequency region are compared in this regard. It is evident from the data attained that impedance intensity decreases on increasing G concentration, from values in the MΩ scale for the pure PCL scaffold to the kΩ scale for the PCL+G scaffolds. The diameter of the semi-circles in the respective plots decrease exponentially in the order ~207, 9, 6, and 0.5 kΩ, for the PCL+0.75G, PCL+1.5G, PCL+3G, and PCL+6G scaffolds, respectively, indicating an improved electron charge transfer capability in the scaffolds due to increasing G concentration. It is notable that there is a significant reduction in impedance on addition of the lowest amount of G (0.75%) used in the PCL+G scaffold, causing a decrease from the MΩ to the kΩ scale. Similar results have been reported by others, either with PCL and G (or a G-derivative) [9,12] or in other polymers, such as polyurethane loaded with G [11].

It is suggested that, in general, hydrophilic surfaces better support cell adhesion [6,29]. Hydrophilicity potential for the PCL+G scaffolds was examined by contact angle measurements to determine the surface energy and interface tensions of a small water droplet on each scaffold type. It is assumed that the lower the contact angle observed, the higher the hydrophilicity and lower the hydrophobicity of the surface (and vice versa). The average contact angle for the 3D-fabricated pure PCL scaffold created here was 107.22 ± 9.39°. Unalan et al. reported a similar contact angle for pure PCL (104.00 ± 8.00°) [27]. Wang et al. reported a lower contact angle (96.00 ± 1.50°) [6], while Ivanova et al. reported a higher contact angle (123 ± 10°) [30]. On increasing the G concentration in PCL+G scaffolds, the mean contact angle was found to decrease, with scaffolds containing higher G concentration (PCL+3G and PCL+6G) showing statistically different contact angle values (98.33 ± 6.75 and 77.56 ± 6.75°), respectively, compared with that for the pure PCL scaffold (107.22 ± 9.39°). Wettability modification of PCL to increase its bioactivity is well-reported; Kumar et al. found that the contact angle of a PCL biofilm was lowered by up to ~12° when modified with up to 5% nanoparticles of graphene oxide (GO), with the same result observed with nanoparticles of amine-modified GO. Seyedsalehi et al. found ~10° lowering of the contact angle of a 3D-printed PCL scaffold when modified with up to 3% reduced GO, and Biscaia et al. found ~20° lowering of the contact angle of a 3D-printed PCL scaffold when modified with 0.5% GNP [31,32,33]. In general, a contact angle above 90° corresponds to a hydrophobic surface, while a contact angle value below 90° corresponds to a hydrophilic surface [6]. Hence, in this study, only the PCL+6G scaffold (77.56 ± 6.75°) is deemed to be truly hydrophilic. As stated previously, scaffolds created from PCL+G mixtures with higher G concentrations (namely, PCL+3G and PCL+6G) had filaments that were ‘rough’, and so for these systems, twelve contact angles measurements were performed to obtain a representative average contact angle.

In vitro cell methods were performed to (1) observe morphology of adhered cells, (2) observe the nuclei of adhered cells, and (3) determine the viability of adhered cells. FESEM images were used to qualitatively confirm the presence of adhered cells on each scaffold type. Images show that cells were visibly adhered to all scaffold types at day 3 with their size, features, and morphology in agreement with those in previous literature reports [15]. Fluorescence microscopy images of stained nuclei were used to qualitatively confirm the presence of adhered cells on each scaffold type. Blue-stained nuclei were visibly adhered to all scaffold types at day 3, confirming cell adherence per scaffold type like that of the FESEM images. Whereas mean nuclei count per scaffold type would normally be possible, the undulating roughened surface topography of the extruded PCL and PCL+G filaments made this less reliable here, as it was not possible to attain consistent focus across the whole focal field of view. Neither the FESEM images nor fluorescence micrographs demonstrate any marked difference in cell numbers on each scaffold type; however, quantification of the cell viability data from the Alamar Blue™ HS test does. The Alamar Blue™ HS test demonstrates that viable cells were present on all scaffold types at days 1, 2, and 3. Importantly, the PCL+0.75G, PCL+1.5G, and PCL+3G scaffolds showed higher fluorescence than that for the pure PCL scaffold. Moreover, the PCL+0.75G and PCL+1.5G scaffolds demonstrated the highest degree of statistical significance (*p* < 0.0001) at days 1, 2, and 3, with the PCL+3G scaffold demonstrating statistical significance at days 1 and 3 (*p* < 0.05 and *p* < 0.001, respectively). The data demonstrate that for the duration of this study (3 culture days), cell viability was improved on the addition of 0.75, 1.5, and 3% (*w*/*w*) G to PCL.

The work presented here demonstrates that 3D scaffolds comprising PCL+0.75G, PCL+1.5G, PCL+3G, and PCL+6G can be successfully fabricated by a simple, rapid, and solvent-free FDM 3D printing process. The introduction of graphene (G) nanoplatelets seeks to provide attributes of hydrophilicity and electrical conductivity to polycaprolactone (PCL) in a manner that promotes their ability to support electrically responsive cells. Results here clearly demonstrate that across the G gradient (0–6%), the scaffolds became less hydrophobic, and even with the smallest addition of G to PCL (0.75%), a marked improvement in both electroactivity and bioactivity was elicited over that of pure PCL.

## 5. Conclusions

Overall, the results obtained here indicate that 1.5% and 3% (*w*/*w*) G-loaded PCL scaffolds, produced via a simple, rapid, and solvent-free FDM 3D printing regime, are promising electrically receptive scaffolds. Given the favourable in vitro results obtained for these samples (in combination with wettability and electrical conductivity measurements), these scaffolds are prime candidates for supporting electrically responsive cells and have potential use in an in vitro tissue model system. It is acknowledged that further physico-chemical, electrical, mechanical, and in vitro characterisations of the scaffolds are required to understand their full potential, along with investigations of any impact of changing print architecture and surface topography on biological response. However, it is clear that this work contributes to the knowledge base and primes future research for adding more attributes of biomimicry to scaffolds to better represent key aspects of real tissues as well as for the creation of new tissue models to study physiological events and diseases and to test new interventions.

## Data Availability

The data presented in this study are available on request from the corresponding author.
